# Association between prehospital arterial hypercapnia and mortality in acute heart failure: a retrospective cohort study

**DOI:** 10.1186/s12873-021-00527-y

**Published:** 2021-11-06

**Authors:** Mathias Fabre, Christophe A. Fehlmann, Kevin E. Boczar, Birgit Gartner, Catherine G. Zimmermann-Ivol, François Sarasin, Laurent Suppan

**Affiliations:** 1 Division of Emergency, Department of Anaesthesiology, Clinical Pharmacology, Intensive Care and Emergency Medicine, Geneva University Hospitals and Faculty of Medicine University of Geneva, Geneva, Switzerland; 2 School of Epidemiology and Public Health, University of Ottawa, Ottawa, Ontario K1G 5Z3 Canada; 3 Ottawa Hospital Research Institute, Ottawa, Ontario ON K1Y 4E9 Canada; 4 University of Ottawa Heart Institute, Ottawa, Ontario ON K1Y 4E9 Canada; 5 Division of Medicine Laboratory, Department of Diagnostics, Geneva University Hospitals and Faculty of Medicine University of Geneva, Geneva, Switzerland

## Abstract

**Background:**

Acute Heart Failure (AHF) is a potentially lethal pathology and is often encountered in the prehospital setting. Although an association between prehospital arterial hypercapnia in AHF patients and admission in high-dependency and intensive care units has been previously described, there is little data to support an association between prehospital arterial hypercapnia and mortality in this population.

**Methods:**

This was a retrospective study based on electronically recorded prehospital medical files. All adult patients with AHF were included. Records lacking arterial blood gas data were excluded. Other exclusion criteria included the presence of a potentially confounding diagnosis, prehospital cardiac arrest, and inter-hospital transfers. Hypercapnia was defined as a PaCO_2_ higher than 6.0 kPa. The primary outcome was in-hospital mortality, and secondary outcomes were 7-day mortality and emergency room length of stay (ER LOS). Univariable and multivariable logistic regression models were used.

**Results:**

We included 225 patients in the analysis. Prehospital hypercapnia was found in 132 (58.7%) patients. In-hospital mortality was higher in patients with hypercapnia (17.4% [23/132] versus 6.5% [6/93], *p* = 0.016), with a crude odds-ratio of 3.06 (95%CI 1.19–7.85). After adjustment for pre-specified covariates, the adjusted OR was 3.18 (95%CI 1.22–8.26). The overall 7-day mortality was also higher in hypercapnic patients (13.6% versus 5.5%, *p* = 0.044), and ER LOS was shorter in this population (5.6 h versus 7.1 h, *p* = 0.018).

**Conclusion:**

Prehospital hypercapnia is associated with an increase in in-hospital and 7-day mortality in patient with AHF.

## Background

Acute Heart Failure (AHF) is a common and life-threatening medical condition which requires urgent evaluation and management and is associated with a high burden of morbidity and mortality [1, 2]. In Switzerland, AHF led to the death or to the readmission of 8120 patients in 2005 [3]. The in-hospital mortality varies from 4 to 11%, and readmission within the next 3 months is frequent, with rates varying between 9 and 30% [4–10].

The treatment of AHF includes vasodilators medication, as well as diuretics and oxygen [1]. Positive end-expiratory pressure, with or without pressure support, has also been shown to be beneficial in case of severe AHF [11–13]. In the prehospital setting, NIV has been shown to reduce intubation rate in AHF [14]. It also seems to alleviate symptoms in the emergency room (ER) [15].

Some factors negatively affect the prognosis of AHF patients including low systolic arterial blood pressure [16], ischemic or parainfectious etiologies [17], renal dysfunction, and polymorbidity [9]. Other negative factors have been described like glucose variation during hospitalization [18], and multiples scores have been created to estimate the AHF-associated prognosis by taking these elements into account [19, 20].

Hypercapnia is common in AHF, with rates varying between 22 and 30% in the in-hospital and emergency setting [21, 22], and reaching up to 58% in the prehospital setting [23]. In this latter setting, hypercapnia has been shown to be associated with a higher rate of intensive care and high-dependent unit admission [23]. This might be linked to the increased need for non-invasive ventilation (NIV) and endotracheal intubation (ETI) described in AHF patients who are hypercapnic at hospital admission [21].

An association between hypercapnia and in-hospital mortality cannot be ruled out, but there is currently little data to support it. The goal of this study was to determine whether prehospital hypercapnia was associated with mortality in AHF.

## Methods

### Study design

This was a retrospective cohort study, based on data obtained from an electronic chart review, exploring the association between prehospital hypercapnia and mortality. The study protocol was approved by the institutional ethics committee of Geneva, Switzerland (project ID 2019–01559, amended 12.11.2019). Patient consent was waived by this committee.

The methods are similar to those used in a previous work [23], the aim of which was to determine the presence of an association between prehospital hypercapnia and admission in intensive care or high-dependency units. Thus, even though the population and outcomes are different, the methods used to collect the data are analogous.

### Setting

This study was carried out using data from the prehospital medical mobile unit (called SMUR for Service Mobile d’Urgence et de Réanimation) of the Geneva University Hospitals in Switzerland. This covers a population of 500′000 persons. This physician-staffed prehospital system has already been described in prior studies [14, 23]. Briefly, a SMUR unit is called whenever a critical situation, such as severe AHF, is identified. These units can either be dispatched by the emergency medical call center or requested by the paramedics already on site. The paramedics and physicians who staff this unit are trained to provide prehospital NIV (defined as application of bilevel positive airway pressure) and perform arterial blood gas (ABG) analysis [23]. Even though venous blood gas analysis could also be considered to assess capnia [24], the current prehospital treatment protocol requires arterial blood to be drawn if prehospital physicians decide to perform an analysis of blood gases. In our system, ABG analysis is strongly encouraged whenever NIV is initiated in the prehospital setting and physicians are only allowed one attempt at drawing arterial blood, in one minute at most.

### Participants

Electronic charts of all patients for whom an intervention took place between July 1, 2016 to January 31, 2020 were screened for inclusion. Prehospital interventions performed after this latter date were excluded to avoid any potential bias that could have been introduced by the COVID-19 pandemic.

Only 126 different diagnoses can be coded in the SMUR’s prehospital medical charts, and only two relate to AHF: “acute pulmonary edema” (APE) and “heart failure” (HF). All patients 18 years or older in whom any of these two diagnoses was coded were screened for inclusion. Patients for whom a diagnosis of APE was coded were automatically included. All patients with a diagnosis of HF were then manually screened to include patients who did present with an acute component to their HF. The ESC definition of AHF i.e., “a rapid onset or worsening of HF symptoms threatening life” [1], was used to determine whether an acute component was present. Any uncertainty regarding diagnosis and inclusion of a patient was settled by consensus between MF and two of the other authors (CAF and LS). All patients for whom prehospital arterial ABG could not be obtained were excluded. The other exclusion criteria were cardiac arrest before or upon SMUR team’s arrival, presence of a mixed diagnosis (such as a concomitant acute chronic obstructive pulmonary disease exacerbation) described by the physician in charge, secondary transfer from another hospital or emergency care structure, and patients who were left on site without transportation to a hospital (and for whom information about mortality was therefore unavailable).

### Data collection

All electronically recorded relevant data were automatically extracted to a Microsoft Excel file (Microsoft Corporation, Redmond, Washington, USA). These data were then imported to an electronic case report form (CRF) created under REDCap (Vanderbilt University, Nashville, Tennessee, USA). All of the data which could not be automatically extracted were manually retrieved and entered into this CRF.

### Exposure and outcomes

Hypercapnia (defined as a PaCO_2_ equal to or higher than 6.0 kPa or 45 mmHg) was the main predictor. The primary outcome was in-hospital mortality. Secondary outcomes were 7-day mortality and ER length of stay (LOS).

Week-end interventions were those performed on Saturdays and Sundays. Night interventions occurred between 7 PM and 7 AM. Length of intervention was the time elapsed between arrival on site and arrival at the hospital.

### Statistical analysis

All statistical analyses were performed using Stata 16 (StataCorp LLC, College Station, Texas, USA). Values are presented as mean and standard deviation (SD) or median and interquartile range (IQR), based on visual inspection of the normality of the distribution. Patients with and without hypercapnia were compared using either the Student t-test, the Wilcoxon–Mann–Whitney test or the Chi2 test, as appropriate. The outcomes for each group were then tabulated. For the primary outcome, univariable logistic regression was performed to compute the crude odds ratio (OR) for the association between hypercapnia and in-hospital mortality. Exploratory multivariable analyses were then performed, adjusting for pre-specified potential confounders, and respecting a ratio of 7-to-10 events per degree of freedom. Confounders were selected based on the literature and physiological plausibility. The adjusted OR with its 95% confidence interval is reported. Finally, the crude association between PaCO_2_ and in-hospital mortality was modelled using restricted-cubic splines and this result was presented graphically. For secondary outcomes, the outcomes were also tabulated. Associations were then tested using a Chi [2] test for the 7-day mortality and the Wilcoxon-Mann-Whitney test for the ER LOS, as this variable was positively skewed. For all tests, a *p*-value lower than 5% was considered statistically significant. Exploratory analyses looking at effect modification by sex were performed using stratification and computing the Mantel–Haenszel statistic.

As the proportion of missing data was extremely low (< 1%) for all vital signs but diastolic blood pressure (29%), lack of data was deemed random and multiple imputation by chained equations was therefore performed, leading to the creation of 10 data sets. All baseline characteristics were used in the imputation model. When not specifically detailed in the medical charts, the Glasgow Coma Scale (GCS) score was assumed to be 15. For the secondary mortality outcome, patients who were discharged alive before 7 days were considered alive at day 7 if no other information regarding their vital status was available on their record.

Assuming an overall mortality of 15%, it was estimated that 192 patients would be sufficient to show a 3-fold increase in mortality in the hypercapnic group with a power of 80% and an alpha error of 5%.

## Results

**Fig. 1:**
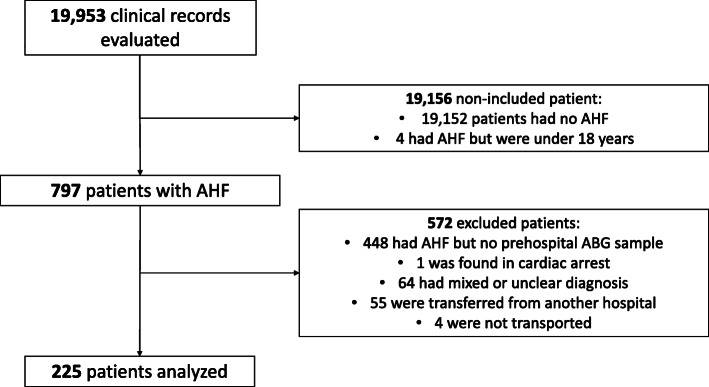
Flowchart

The SMUR provided care to a total of 19,953 patients during the study period, 797 of whom met the inclusion criteria. After excluding 572 patients (Fig. 1) 225 patients (28.2%) were included in the analysis. The main exclusion criteria was a lack of ABG (448 patients). The rationale for no ABG being performed was unknown in most cases (291/448, 65%). Documented reasons for absence of ABG were a failed attempt by physician or collection of a venous sample (78), technical failure of the analyzer (31), medical decision (45), and patient refusal [3].

Baseline patient characteristics are described in Table 1. Overall, patients had a mean age of 83.9 years (with 27.6% of them being 90 years or older), and 58.7% of them had prehospital hypercapnia. Patients without hypercapnia had a median PaCO_2_ of 5.1 kPa (IQR: 4.7–5.6), compared to 7.4 kPa (IQR: 6.6–8.9) for patients with hypercapnia. Most baseline characteristics were otherwise similar between groups. Female patients, which represented 63.6% of the population, were more likely to be hypercapnic (69.7% versus 54.8%, *p* = 0.023). Hypercapnic patients had higher diastolic blood pressures (103.6 mmHg versus 93.7 mmHg, *p* = 0.036) and were more likely to have abnormal GCS scores (62.1% had a normal GCS versus 81.7%, *p* = 0.002). They were also more likely to be treated by NIV in the field (93.9% versus 74.2%, *p* < 0.001).

**Table 1:** Baseline characteristics^1^

	All patients (*n* = 225)	No prehospital hypercapnia (*n* = 93)	Prehospital hypercapnia (*n* = 132)	*p*-value
Age (y) – median (IQR)	86 (78–90)	86 (77–90)	86 (79–90)	0.554
Sex (f) – n (%)	143 (63.6)	51 (54.8)	92 (69.7)	0.023
Therapeutic limitation – n (%)	113 (50.2)	44 (47.3)	69 (52.3)	0.464
Week-end intervention – n (%)	70 (31.1)	34 (36.6)	36 (27.7)	0.138
Night intervention – n (%)	114 (50.67)	41 (44.1)	73 (55.3)	0.245
Length of intervention (min) – median (IQR)	39 (34–47)	42 (35–47)	38 (34–46.5)	0.187
Personal history – n (%)				
*Hypertension*	190 (84.4)	74 (79.6)	116 (87.9)	0.090
*Coronary heart disease*	77 (34.2)	37 (39.8)	40 (30.3)	0.140
*Atrial fibrillation*	100 (44.4)	54 (40.9)	46 (49.5)	0.204
*Pacemaker*	41 (18.2)	19 (20.4)	22 (16.7)	0.471
*Active cancer*	14 (6.2)	5 (5.4)	9 (6.8)	0.659
*Chronic obstructive pulmonary disease*	30 (13.3)	12 (12.9)	18 (13.6)	0.873
*Diabetes mellitus*	60 (26.7)	23 (24.7)	37 (28.0)	0.582
*Chronic renal failure*	114 (50.7)	45 (48.4)	69 (52.3)	0.567
*Prior hospitalisation for heart failure*	93 (41.3)	33 (35.5)	60 (45.5)	0.135
*Tobacco*	26 (11.6)	10 (10.8)	16 (12.1)	0.752
Prehospital vital signs				
*Heart rate (1/min) – mean ± SD*	108.2 ± 23.3	105.3 ± 24.1	110.2 ± 22.5	0.123
*Systolic blood pressure (mmHg) – mean ± SD*	172.8 ± 33.1	167.7 ± 32.0	176.4 ± 33.5	0.052
*Diastolic blood pressure (mmHg) – mean ± SD*	99.2 ± 21.8	93.7 ± 18.8	103.6 ± 23.0	0.036
*Respiratory rate (1/min) – mean ± SD*	36.0 ± 7.6	35.2 ± 8.1	36.5 ± 7.3	0.884
*Oxygen saturation (%) – median (IQR)*	88 (80–93)	88 (80–91)	88 (78.5–95)	0.624
*Oxygen saturation < 95% – n (%)*	175 (77.8)	80 (86.0)	95 (72.0)	0.013
*Oxygen saturation < 90% – n (%)*	141 (62.7)	60 (64.5)	81 (61.4)	0.630
*GCS = 15 – n (%)*	158 (70.2)	76 (81.7)	82 (62.1)	0.002
Prehospital PaCO_2_ (kPa) – median (IQR)	6.2 (5.3–7.7)	5.1 (4.7–5.6)	7.4 (6.6–8.9)	< 0.001
Prehospital pH – median (IQR)	7.31 (7.25–7.38)	7.38 (7.32–7.42)	7.26 (7.20–7.31)	< 0.001
Prehospital bicarbonate – median (IQR)	24 (21–28)	22.0 (19.7–24.1)	26.2 (22.5–30.5)	< 0.001
Prehospital NIV – n (%)	193 (85.8)	69 (74.2)	124 (93.9)	< 0.001
Laboratory values				
*Creatinine (*μmol*/l) – median (IQR)*	112 (81–146)	114 (84–151)	108 (80–143)	0.364
*Pro-BNP (pg/ml)– median (IQR)*	3583 (1834–8847)	4085 (2000–12,220)	3322 (1801–6722)	0.393

^1^ *SD* standard deviation, *IQR* Interquartile range, *NIV* Non-invasive ventilation, *GCS* Glasgow coma scale, *pro-BNP* pro-brain natriuretic peptide. Therapeutic limitation was defined as “Advanced care planning preventing intensive care unit admission”

**Fig. 2:**
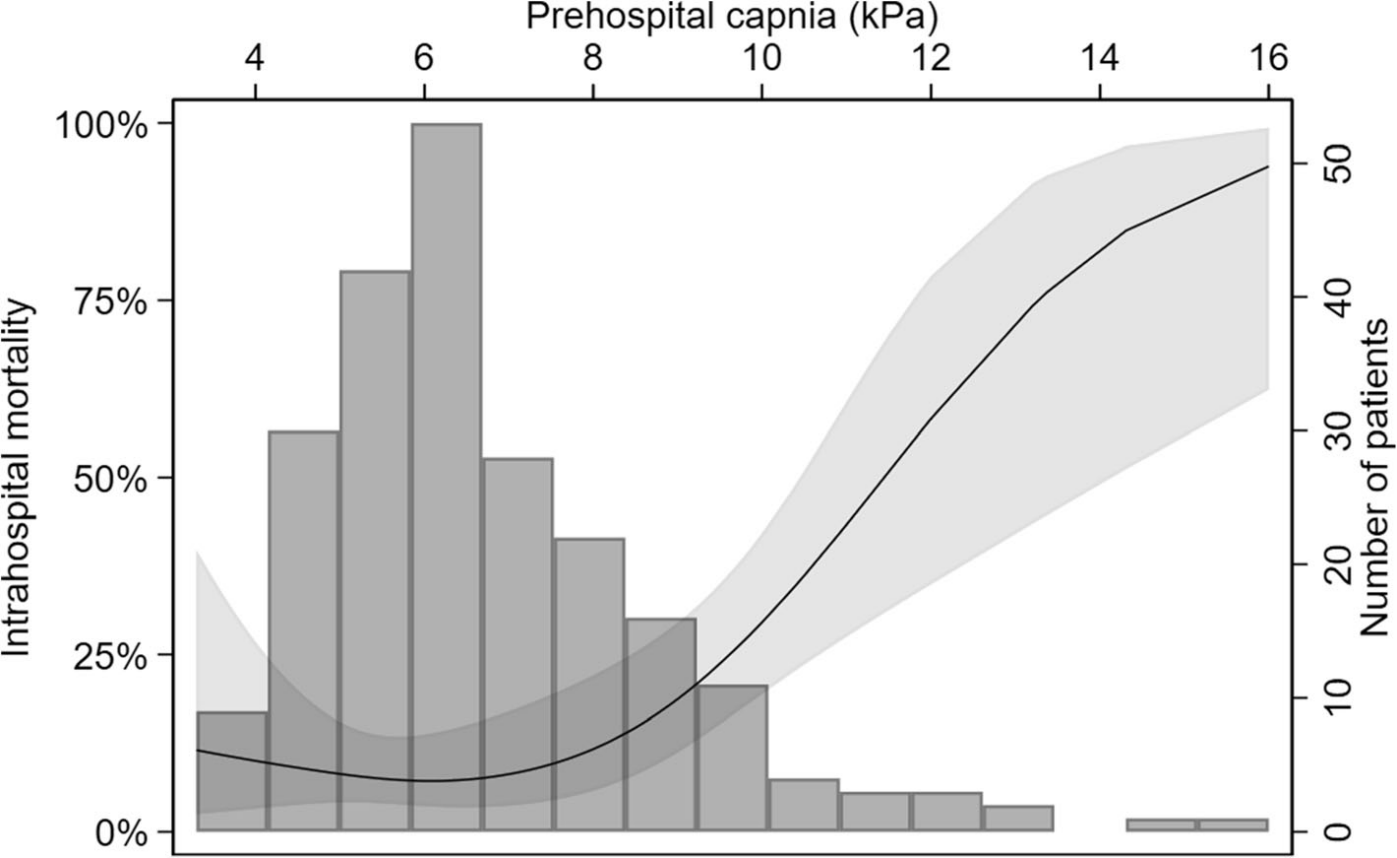
Crude association between PaCO2 and intrahospital mortality, with patients repartition

The overall in-hospital mortality was 12.9% and was significantly higher in patients with prehospital hypercapnia than in those without (17.4% versus 6.5%, *p* = 0.016), with an unadjusted OR of 3.06 (95%CI 1.19–7.85) (Table 2). After adjustment for history of chronic renal failure, chronic obstructive pulmonary disease and hypertension, the adjusted OR was 3.18 (95%CI 1.22–8.26) (Table 3). Figure 2 presents the association between in-hospital mortality and prehospital PaCO_2_, and shows a dose-dependent association. Finally, although in-hospital mortality was slightly higher in male patients than in female patients (17.1% versus 10.5%, *p* = 0.156), the association between prehospital hypercapnia and the outcomes did not differ by sex (*p* = 0.810).

**Table 2:** Outcomes^1^

	All patients (*n* = 225)	No prehospital hypercapnia (*n* = 93)	Prehospital hypercapnia (*n* = 132)	*p*-value
**Primary outcome**				
In-hospital mortality – n (%)	29 (12.9)	6 (6.5)	23 (17.4)	0.016
**Secondary outcomes**				
7-day mortality – n (%)	23 (10.2)	5 (5.4)	18 (13.6)	0.044
*ER Length of stay (h) – median (IQR)*	6.0 (4.3–9.2)	7.1 (4.5–11.4)	5.6 (4.0–8.4)	0.018

^1^ *ER* emergency room, *IQR* interquartile range

**Table 3:** Univariable and multivariable logistic regression for in-hospital mortality^1^

	Univariable analysis		Multivariable analysis	
	cOR	95%CI	aOR	95%CI
Hypercapnia	3.06	1.19–7.85	3.18	1.22–8.26
COPD	1.05	0.34–3.25	1.06	0.33–3.37
Hypertension	0.87	0.31–2.45	0.63	0.21–1.92
Chronic renal failure	1.45	0.66–3.19	1.53	0.67–3.52

^1^ *cOR* crude odd ratio, *aOR* adjusted odd ratio, *CI* confidence interval

There was also a significant difference in 7-day mortality (13.6% vs 5.5%, *p* = 0.044), with an unadjusted OR of 2.68 (95%CI 1.69–4.24). The median ER length of stay was 6.0 h and was longer for patients without hypercapnia than for patients with hypercapnia (7.1 versus 5.6, *p* = 0.018) (Table 2).

## Discussion

This cohort study shows that prehospital hypercapnia is associated with an increase in in-hospital and 7-day mortality in patients with AHF. A “dose-dependent” association between the value of PaCO_2_ and mortality was also observed.

Even though association does not equal causation, these results are strengthened by physiologic plausibility, a dose-dependent association, and similar results reported in in-hospital studies [21]. Indeed, a prospective cohort study of patients with acute respiratory distress syndrome reported a similar association between hypercapnia and in-hospital mortality [25]. In addition, animal models also support this link, as hypercapnia was found to be associated with a reduced probability of pulmonary wound repair in a rat model of ARDS [26].

Hypercapnic patients were more likely to present with higher systolic and diastolic blood pressures than normocapnic patients. Theoretically, higher blood pressures could potentially contribute to an increased risk of pulmonary edema and thus a decrease of CO_2_ clearance and oxygen uptake, a pathophysiologic process that has already been theorized to explain hypercapnia in AHF [27]. The resulting hypoxemia could therefore contribute to the increased mortality rate. Another hypothesis is that higher blood pressure are found in sicker patients given their higher sympathetic stress.

This study reports a higher proportion of hypercapnic patients compared to in-hospital studies, as well as a higher rate of hypoxemic patient [21, 22]. Even though some initially hypercapnic patients might have normalized their PaCO_2_ and PaO_2_ after prehospital treatment, a selection bias cannot be ruled out. Indeed, the SMUR units are only called to attend to the sickest patients with AHF, while patients with milder symptoms can be managed by paramedics only. The subset of patients examined in the present study is rather limited, and most patients admitted in the ED for heart failure are not breathless at rest [28]. Nevertheless, patients with AHF taken care of by prehospital teams deserve special consideration as they are usually critically ill and therefore more likely to require advanced therapies. This theory is supported by the overall mortality rate which was also slightly higher than the rates reported in larger cohort studies [5, 9].

Normocapnic patients spent more time in the ER than hypercapnic patients, an effect already observed when only considering patients who were eligible for admission in an intensive care or high-dependency unit [23]. The most likely explanation is that given the worse clinical status of these patients, clinicians can rapidly decide upon admission disposition and therefore need less time and data to make their admission decision. Thus, prehospital PaCO_2_ could be used as a tool to improve patient disposition and reduce the time spent in the ER. Further prospective trials, which could compare ER LOS depending on the systematic analysis of prehospital ABG, could be designed to investigate the clinical applicability of this finding.

This study has several limitations. First, as already mentioned above, SMUR units are probably called to help manage the sickest of AHF patients, thereby inducing potential selection bias. Another potential selection bias is related to the local AHF treatment protocol, which strongly recommends performing ABG analysis whenever NIV is considered, but also acknowledges that physicians should not waste time before initiating treatment. Therefore, several AHF patients who would otherwise have met the inclusion criteria could not be analysed because an ABG was not performed. Consequently, even though the population should not be markedly different, the risk of bias still exists. Another limitation is the lack of information regarding the fraction of inspired oxygen when ABG was performed. This potential confounder could not be assessed in our study given its unreliable reporting in the prehospital medical file. We were also prevented from determining the effect a normalization (or even a decrease) of PaCO_2_ might have on mortality as in-hospital ABG analysis was missing in 33% of all analysed cases. Other potential variables of interest, such as volume status, left ventricular ejection fraction, and troponin were not reported as they were inconsistently recorded and as the high proportion of missing data would have prevented their interpretation. In addition, although the study was adequately powered to show a difference in mortality, the sample size was too small to adjust for many covariates. Therefore, only the most clinically relevant covariates were chosen for adjustment, and residual confounding resulting in overestimated or underestimated results cannot be excluded. Even though usual, prehospital and in-hospital medication could have influenced the patients’ outcome, such data was not straightforward to retrieve and its reliability would have been limited. We therefore refrained from extracting and including this data, which would have been difficult to analyse given the limited sample size. Even though there is little reason to believe that different treatments would have been given according to the prehospital PaCO_2_ values, a bias linked to differences in the choice of medications cannot be formally excluded. Likewise, the analyses regarding effect modification by sex should be interpreted with caution given the small sample size. Finally, the generalization of these results is limited by the fact that this was a monocentric study performed in a supervised and physician-staffed prehospital system. Due to the exclusion of respiratory distress aetiologies other than AHF, our findings cannot be transposed to other populations.

Nevertheless, this study also has several strengths. First, the use of automatically extracted data guarantees, at least in part, its validity. Second, the originality of the analysis, including the use of restricted cubic splines to model the association between PaCO_2_ and mortality in addition to the more traditional approach using a usual cut-off, improves the presentation and interpretability of the results. Indeed, binarization or categorization of continuous variables assumes that there is a discontinuity in response, which is extremely unlikely.

This study should encourage further research on the topic, and the actual clinical utility of prehospital ABG analysis should be assessed by means of a randomised controlled trial. Moreover, a multi-centre study with a bigger sample size would allow to adjust for more covariates. Finally, should the added value of prehospital ABG be confirmed, specific interventions could be devised to increase the utilization of the ABG in a pre-hospital setting.

## Conclusion

Prehospital hypercapnia is associated with an increase in in-hospital and 7-day mortality in patients with AHF. Prospective randomized studies should be performed before systematic prehospital analysis can be recommended in these patients.

## Data Availability

The datasets used and analysed during the current study are available from the corresponding author on reasonable request.

## Abbreviations

ABG: Arterial Blood Gas
AHF: Acute Heart Failure
APE: Acute pulmonary Edema
ARDS: Acute Respiratory Distress Syndrome
CRF: Case Report Form
ER: Emergency Room
ETI: Endotracheal Intubation
GCS: Glasgow Coma Scale
HF: Heart Failure
LOS: Length of Stay
NIV: Non-Invasive Ventilation
OR: Odds Ratio
SMUR: Service Mobile d’Urgences et de Réanimation
